# Pravastatin Protects Cytotrophoblasts from Hyperglycemia-Induced Preeclampsia Phenotype

**DOI:** 10.3390/cells13181534

**Published:** 2024-09-13

**Authors:** Ahmed F. Pantho, Sara Mohamed, Janhavi V. Govande, Riddhi Rane, Niraj Vora, Kelsey R. Kelso, Thomas J. Kuehl, Steven R. Lindheim, Mohammad N. Uddin

**Affiliations:** 1 Artemis Biotechnologies LLC, Temple, TX 76504, USA; ahmed.pantho@artemisbiotech.org (A.F.P.); tjkuehl@peoplepc.com (T.J.K.); 2 Baylor Scott & White Health, Temple, TX 76508, USA; sara.mohamed@bswhealth.org (S.M.); niraj.vora@bswhealth.org (N.V.); kelsey.kelso@bswhealth.org (K.R.K.); steven.lindheim@bswhealth.org (S.R.L.); 3 University of Texas Medical School at Houston, Houston, TX 77030, USA; janhavi.v.govande@uth.tmc.edu; 4 Texas A&M University College of Medicine, College Station, TX 77807, USA; riddhirane@tamu.edu

**Keywords:** preeclampsia, hyperglycemia, cytotrophoblast, pravastatin, migration

## Abstract

There are no effective therapies to prevent preeclampsia (PE). Pravastatin shows promise by attenuating processes associated with PE such as decreased cytotrophoblast (CTB) migration, aberrant angiogenesis, and increased oxidative stress. This study assesses the effects of pravastatin on hyperglycemia-induced CTB dysfunction. Methods: Human CTB cells were treated with 100, 150, 200, 300, or 400 mg/dL glucose for 48 h. Some cells were pretreated with pravastatin (1 µg/mL), while others were cotreated with pravastatin and glucose. The expression of urokinase plasminogen activator (uPA), plasminogen activator inhibitor 1 (PAI-1) mRNA, vascular endothelial growth factor (VEGF), placenta growth factor (PlGF), soluble fms-like tyrosine kinase-1 (sFlt-1), and soluble endoglin (sEng) were measured. CTB migration was assayed using a CytoSelect migration assay kit. Statistical comparisons were performed using an analysis of variance with Duncan’s post hoc test. Results: The hyperglycemia-induced downregulation of uPA was attenuated in CTB cells pretreated with pravastatin at glucose levels > 200 mg/dL and cotreated at glucose levels > 300 mg/dL (*p* < 0.05). Hyperglycemia-induced decreases in VEGF and PlGF and increases in sEng and sFlt-1 were attenuated in both the pretreatment and cotreatment samples regardless of glucose dose (*p* < 0.05). Pravastatin attenuated hyperglycemia-induced dysfunction of CTB migration. Conclusions: Pravastatin mitigates stress signaling responses in hyperglycemic conditions, weakening processes leading to abnormal CTB migration and invasion associated with PE in pregnancy.

## 1. Introduction

Preeclampsia (PE) is a hypertensive disorder in pregnancy with multiple etiological factors that currently affects 3–8% of pregnancies worldwide [1,2,3]. Women diagnosed with diabetes mellitus before pregnancy have an increased risk of developing PE than non-diabetic women (approximately 1 in 5 vs. 1 in 20, respectively) [4,5]. At the early stage of pregnancy, paternal genetic factors may alter the implantation and vascularization of fetoplacental unit that leads to PE [6]. The PE cases were significantly associated with higher body mass index (BMI) before and after pregnancy, shorter gestational age (SGA) at delivery and higher levels of high-temperature requirement factor A1 (HtrA1) values compared to healthy individuals [7]. The pathophysiology of PE is not yet well understood. However, multiple mechanisms associated with placental formation have been implicated in the disease progression [8]. Those mechanisms include decreased cytotrophoblast (CTB) cell migration and invasion, alterations in angiogenic formation, and increased apoptotic signaling, all of which result in abnormal placement, structure, and function of the placenta [9]. CTB cells are essential in pregnancy development. During the first trimester, extravillous CTB cells invade the myometrium and decrease spiral artery resistance, promoting maternal blood flow toward the placenta [10]. If this process is disrupted, adequate blood flow is not established and the shallow placentation that results increases the likelihood of PE development [11]. A recent study showed that hyperglycemia disrupts proper CTB function, thereby indicating a possible link between diabetes mellitus and the development of PE [12].

Endometrial invasion by CTB cells is facilitated with the digestion of extracellular matrix by proteinase activation of the plasmin pathway [13]. Urokinase plasminogen activator (uPA) is a protease found in proliferating CTB cells and plays a vital role in CTB cell adhesion and invasion [14,15]. Plasminogen activator inhibitor-1 (PAI-1) is localized to CTB cells in contact with maternal tissue, thereby facilitating the development of the maternal–fetal interface [16,17]. The p-38 mitogen-activated protein kinase (MAPK) responds to cellular stress and regulates CTB proliferation, differentiation, invasiveness, and apoptosis [18,19,20,21,22,23]. Proliferating cell nuclear antigen (PCNA) is a proliferation marker mainly expressed in villous and invasive CTB cells in the placenta [24]. Studies have shown that in PE, PCNA expression is decreased in decidual and basal cells and increased in villous cells [24].

Angiogenic factors such as vascular endothelial growth factor (VEGF) and placental growth factor (PlGF) are expressed during placental development. Both are downregulated in hyperglycemic conditions. Conversely, antiangiogenic factors such as soluble FMS-like tyrosine kinase (sFLT) and soluble endoglin (sENG) are upregulated in increased glucose concentrations [12].

Due to the pathogenic similarity between PE and cardiovascular disease, 3-hydroxy-3-methyl-glutaryl-coenzyme A reductase inhibitors, or statins, are being studied as potential prophylactic treatment for PE [25,26,27,28,29,30]. In previous studies, statins have been shown to disrupt the mechanisms that contribute to the development of PE, such as angiogenic instability and increases in pro-inflammatory markers [25]. Pravastatin has been chosen to be studied because it poses minimal risk of transplacental exposure to the fetus, given its low passive diffusion, hydrophilicity, and relative efflux by placental transporters [31,32,33]. It achieves maximum plasma levels 1–1.5 h after oral administration and has an elimination half-life of 1.77 h [31].

However, pravastatin still holds some safety concerns; it currently holds a Food and Drug Administration (FDA) Pregnancy Category X designation. Category X describes medications for which “studies in animals or humans have demonstrated fetal abnormalities and/or there is positive evidence of human fetal risk based on adverse reaction data from investigational or marketing experience, and the risks involved in the use of the drug in pregnant women outweigh potential benefits” [34]. When this designation was given, there was no research indicating any benefits of the use of statins in pregnancy. There was concern that inhibiting cholesterol synthesis during embryogenesis could pose a threat to the fetus. However, since then, multiple studies have shown no connection between statin use and unfavorable pregnancy outcomes. One such study analyzed data from two large case–control studies of birth defects (the National Birth Defects Prevention Study and the Slone Epidemiology Center Birth Defects Study) and found no correlation between the use of statins and neonatal abnormalities [35]. Another study by The Medical Genetics Branch of the National Institute of Health reviewed cases of statin exposure in pregnancy reported to the FDA and found no instances of adverse outcomes [36]. A recent pilot safety study investigating maternal–fetal safety data for pravastatin when used as a prophylactic daily treatment during pregnancy showed reassuring results, with no noted adverse effects on maternal or fetal wellbeing [25,34,37].

Due to pravastatin’s favorable pharmacokinetic profile and unsupported teratogenicity claims, it poses itself as a reasonable medication for PE prophylaxis. In previous studies at our institution, our research team demonstrated a correlation between hyperglycemia-induced stress signaling and a disruption of the invasiveness and angiogenic balance of CTB cells [3]. Using a similar methodology for this study, we have now evaluated how the use of pravastatin influences these hyperglycemia-induced effects to determine whether this medication may be an option for PE prophylaxis, particularly in diabetic patients.

## 2. Materials and Methods

### 2.1. Cells

CTB Cell Culture: The human extravillous CTB cell line Sw.71 that was utilized in the present study was derived from first-trimester chorionic villus tissue and was provided by Dr. Gil G. Mor at Yale University School of Medicine, New Haven, CT, USA. These cells are well characterized and possess many similarities to isolated primary cells, including the expressions of cytokeratin-7, HLA class I antigen, HLA-G, BC-1, CD9, human chorionic gonadotropin, and human placental lactogen. The Sw.71 cells were cultured in DMEM/F-12 (Invitrogen, Waltham, MA, USA) supplemented with 10% fetal bovine serum, 10 mM Hepes, 0.1 mM MEM non-essential amino acids, 1 mM sodium pyruvate, and 100 U/mL penicillin/streptomycin. Cells were then incubated at 37 °C, with 5% CO_2_, 8% O_2_, and 99% humidity (Fisher Scientific, Isotemp CO_2_ Incubator, Hampton, NH, USA), with no exposure to hypoxic conditions.

### 2.2. Cell Treatments

Effect of hyperglycemia on CTB Cells: CTB cells were seeded on 6-well plates and were incubated in serum-free media for 24 h. The cells were then treated with 100, 150, 200, 300, or 400 mg/dL of glucose (Sigma-Aldrich, Inc., St. Louis, MO, USA) for 48 h. Some cells were pretreated with 1 µg/mL of pravastatin before treatment with glucose, while others were cotreated with 1 µg/mL of pravastatin and the above glucose levels.

### 2.3. Primers and Reagents

Primers: The RT2 qPCR primer assays for human genes were sourced from QIAGEN (Valencia, CA, USA) and include the following: plasminogen activator, urokinase (uPA), catalog no. PPH00796C-200, Entrez Gene ID 5328; serpin peptidase inhibitor, clade E (PAI-1), catalog no. PPH00215F-200, Entrez Gene ID 5054; glyceraldehyde-3-phosphate dehydrogenase (GAPDH), catalog no. PPH00150F-200, Entrez Gene ID 2597; and actin, beta (β-actin), catalog no. PPH00073G-200, Entrez Gene ID 60.

### 2.4. Real-Time PCR

Real-Time PCR for uPA and PAI-1: Following treatment, the media were carefully removed from the cells, and Lysis/Binding Solution from the Ambion RNAqueous-4PCR Kit (Invitrogen) was added to the cells. The cells were then scraped into tubes, where an equal volume of 64% ethanol was added and mixed thoroughly. The mixture was transferred to a filter and centrifuged into a separate tube. The filter was washed first with Wash Solution #1 and subsequently with Wash Solution #2/3 from the same kit. Preheated Elution Solution was then used to elute the RNA from the filter into tubes. Next, 10 µL of 10× DNase I Buffer and 1 µL of DNase I from the kit were added to the RNA samples, which were incubated at 37 °C for 30 min in a heat block. To inactivate the DNase, 11 µL of DNase Inactivation Reagent from the kit was added, mixed for 2 min, and centrifuged. RNA concentration in each sample was determined using a Nanodrop spectrophotometer (Thermo Fisher Scientific, Waltham, MA, USA). The RNA samples were then heated at 75 °C for 3 min before being placed on ice. In a new tube, 2 µL of Oligo(dT) from the Ambion RETROscript First Strand Synthesis Kit (Invitrogen) was added to 10 µL of RNA. Subsequently, 2 µL of 10× RT Buffer, 4 µL of dNTP Mix, 1 µL of RNase Inhibitor, and 1 µL of M-MLV RT (Moloney Murine Leukemia Virus Reverse Transcriptase; Promega Corporation, Madison, WI, USA) from the kit were added, bringing the total volume to 20 µL. The mixture was gently mixed and briefly centrifuged before being placed in a Perkin Elmer GeneAmp 9600 PCR Thermal Cycler. The tubes were incubated at 42–44 °C for 1 h, followed by incubation at 92 °C for 10 min to inactivate the reverse transcriptase. The concentration of cDNA in each sample was measured using a Nanodrop spectrophotometer. For the real-time PCR, primers for PLAU (uPA), SERPINE1 (PAI-1), glyceraldehyde-3-phosphate dehydrogenase (GAPDH), and ACTB (β-actin) were utilized, all of which were purchased from SABiosciences, (Frederick, MD, USA). iTaq SYBR Green Supermix with ROX (Bio-Rad Laboratories, Hercules, CA, USA) was also used. Each well in the PCR plate contained 1 µL of cDNA, 1 µL of primer, 12.5 µL of SYBR Green, and 10.5 µL of water. The real-time PCR was carried out on a Bio-Rad iCycler iQ5 using a two-step cycling protocol.

### 2.5. Migration Assay

Migration Assay: Cell migration was measured using a CytoSelect Assay (CBA-108; Cell Biolabs Inc., San Diego, CA, USA) as described previously [12,23]. After the CyQuant GR Dye solution was added to the cells, fluorescence was measured at 480 nm/520 nm on a fluorescence plate reader (CytoFluor Series 4000 Fluorescence Multi-Well Plate Reader, Applied Biosystems, Waltham, MA, USA).

### 2.6. Enzyme-Linked Immunosorbent Assay (ELISA)

Enzyme-Linked Immunosorbent Assay for Endoglin (sENG), sFLT-1, Vascular Endothelial Growth Factor (VEGF), and PlGF: After the treatment, the media removed from cells were placed in tubes. Levels of anti-angiogenic (sENG and sFLT-1) and angiogenic (VEGF and PlGF) factors were measured by the commercially available ELISA kits from R&D Systems. For sENG, a Human Endoglin/CD105 Quantikine ELISA Kit (R&D Systems, Minneapolis, MN, USA; Catalog #: DNDG00) was used. For sFLT-1, a Human sVEGF R1/Flt-1 Quantikine ELISA Kit (R&D Systems; Catalog #: DVR100C) was used. For VEGF, a Human VEGF Quantikine ELISA Kit (R&D Systems; Catalog #: DVE00) was used. For PlGF, a Human PlGF Quantikine ELISA Kit (R&D Systems; Catalog #: DPG00) was used.

### 2.7. Statistical Analysis

Statistical Method: Data are expressed as mean ± SE. Statistical significance was assessed by ANOVA and Duncan’s post hoc test for differences between pravastatin pre- or cotreatment on glucose effects. *p* < 0.05 is taken as significant.

## 3. Results

### 3.1. Pravastatin Attenuated the Hyperglycemia-Induced Downregulation of uPA and PAI-1, and mRNA Expression

**Figure 1:**
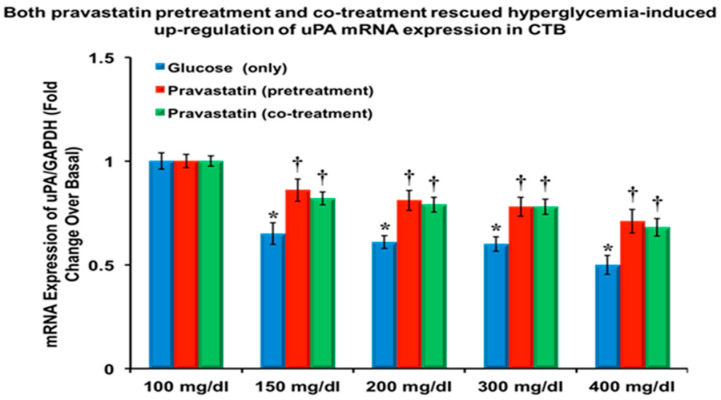
Plot of uPA gene expression for cytotrophoblast cells responding in vitro to hyperglycemia and either pre- or cotreatment with pravastatin. uPA gene expression was increased in CTB cells pretreated with pravastatin at glucose levels > 200 mg/dL, whereas this effect was not seen with cotreatment until >300 mg/dL (*n* = 6, four replicates each; *p* < 0.05). Both * and † are statistically significant.

As shown in Figure 1, the mRNA expression for uPA, standardized to GADPH, is downregulated (*p* = 0.0059) in CTB cells treated with ≥150 mg/dL of glucose compared to basal glucose (100 mg/dL). Figure 1 also shows that pravastatin pretreatment attenuated glucose-induced downregulation of uPA mRNA expression.

**Figure 2:**
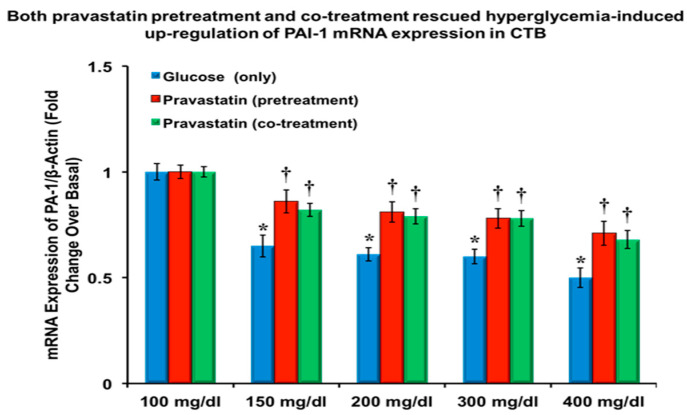
Plot of PAI-1 gene expression relative to GAPDH for cytotrophoblast cells responding in vitro to hyperglycemia and either pre- or cotreatment with pravastatin. PAI-1 gene expression was increased in both the pretreatment and cotreatment samples regardless of glucose dose (*n* = 6, four replicates each; *p* < 0.05). Both * and † are statistically significant.

The graphs in Figure 2 depict that the mRNA expression for PAI-1, normalized to β-actin, is downregulated (*p* = 0.0017) in CTB cells treated with ≥200 mg/dL of glucose compared to basal glucose (100 mg/dL). Figure 2 also demonstrates pravastatin pretreatment attenuating glucose-induced downregulation of PAI-1 mRNA expression.

### 3.2. Pravastatin Attenuated the Hyperglycemia-Induced Reduction in VEGF and PlGF

**Figure 3:**
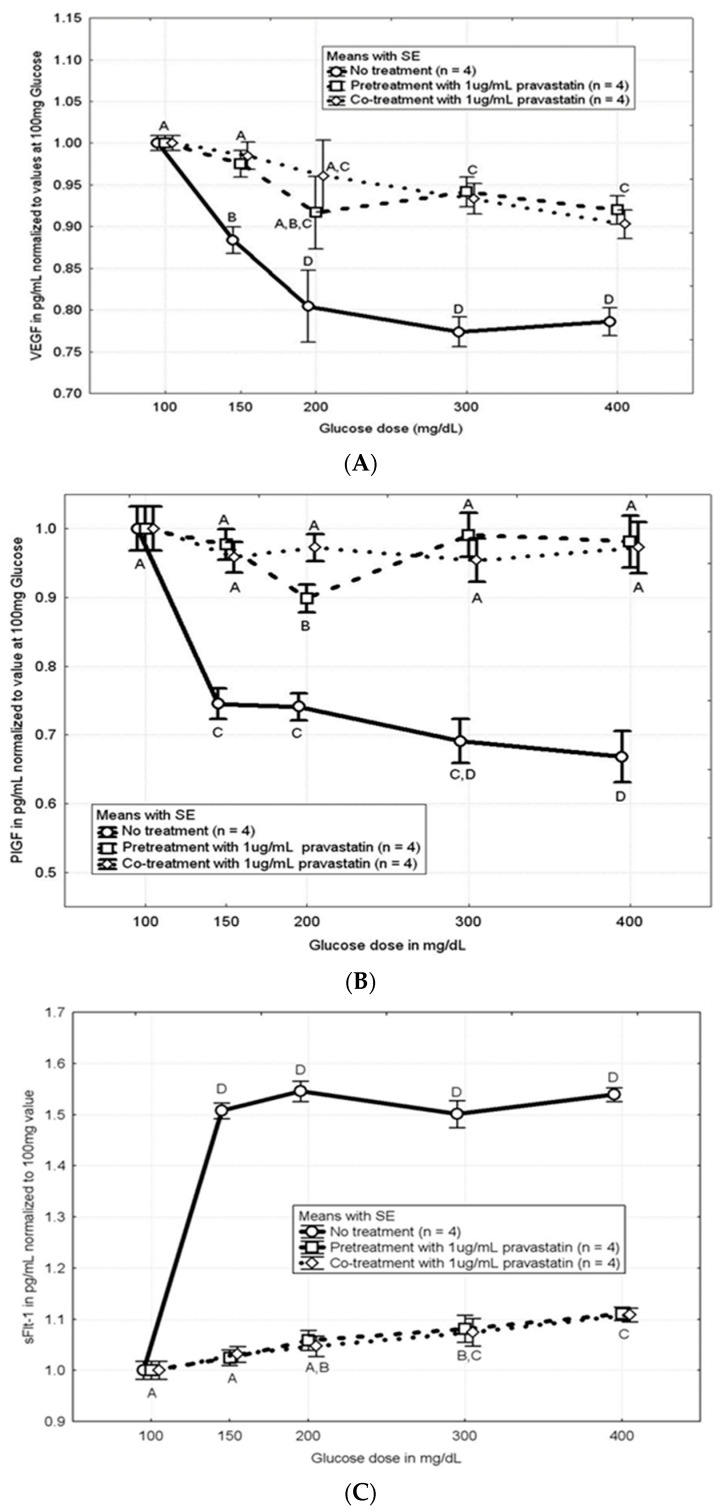

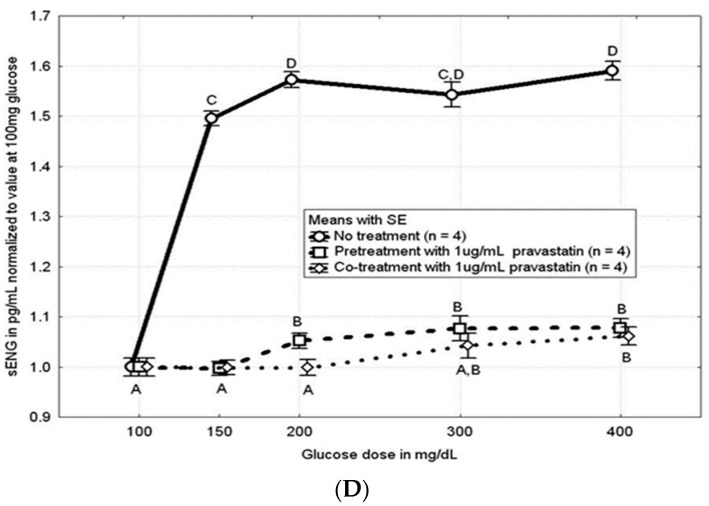
(**A**) Plot of VEGF concentration relative to concentration of VEGF at 100 mg/dL glucose concentration, with CTB cells responding to increasing glucose concentrations and no treatment, cotreatment, or pretreatment with pravastatin. Significant increases in VEGF concentrations were seen for both cotreatment and pretreatment groups at all supraphysiologic glucose levels. Both pravastatin pretreatment and cotreatment rescued CTB cells from hyperglycemia-induced downregulation of VEGF. Treatment groups differ (*p* < 0.001 using ANOVA). Means with different letters differ (*n* = 8, four replicates each; *p* < 0.05 using Duncan’s post hoc test). (**B**) Plot of PlGF concentration relative to concentration of PlGF at 100 mg/dL glucose concentration, with CTB cells responding to increasing glucose concentrations and no treatment, cotreatment, or pretreatment with pravastatin. Significant increases in PlGF concentrations were seen for both cotreatment and pretreatment groups at all supraphysiologic glucose levels. Both pravastatin pretreatment and cotreatment rescued CTB cells from hyperglycemia-induced downregulation of PlGF. Treatment groups differ (*p* < 0.001 using ANOVA). Means with different letters differ (*n* = 8, four replicates each; *p* < 0.05 using Duncan’s post hoc test). (**C**) Plot of sFLT-1 concentration relative to concentration of sFLT-1 at 100 mg/dL glucose concentration, with CTB cells responding to increasing glucose concentrations and no treatment, cotreatment, or pretreatment with pravastatin. Significant decreases in sFLT-1 concentrations were seen for both cotreatment and pretreatment groups at all supraphysiologic glucose levels. Both pravastatin pretreatment and cotreatment rescued CTB cells from hyperglycemia-induced upregulation of sFlt-1. Treatment groups differ (*p* < 0.001 using ANOVA). Means with different letters differ (*n* = 8, four replicates each; *p* < 0.05 using Duncan’s post hoc test). (**D**) Plot of sENG concentration relative to concentration of sENG at 100 mg/dL glucose concentration, with CTB cells responding to increasing glucose concentrations and no treatment, cotreatment, or pretreatment with pravastatin. Significant decreases in sENG concentrations were seen for both cotreatment and pretreatment groups at all supraphysiologic glucose levels. Both pravastatin pretreatment and cotreatment rescued CTB cells from hyperglycemia-induced upregulation of sENG. Treatment groups differ (*p* < 0.001 using ANOVA). Means with different letters differ (*n* = 8, four replicates each; *p* < 0.05 using Duncan’s post hoc test).

Figure 3A,B demonstrate that the hyperglycemia-induced decreases in concentrations of both VEGF and PlGF were attenuated in both the pretreatment and cotreatment samples regardless of glucose dose (*p* < 0.05).

### 3.3. Pravastatin Attenuated the Hyperglycemia-Induced Increase in sFLT and sENG

Figure 3C,D demonstrate that the hyperglycemia-induced increases in concentrations of sFLT-1 and sENG were attenuated in both the pretreatment and cotreatment samples regardless of glucose dose (*p* < 0.05).

### 3.4. Pravastatin Attenuated the Hyperglycemia-Induced Loss of CTB Cell Migration

**Figure 4:**
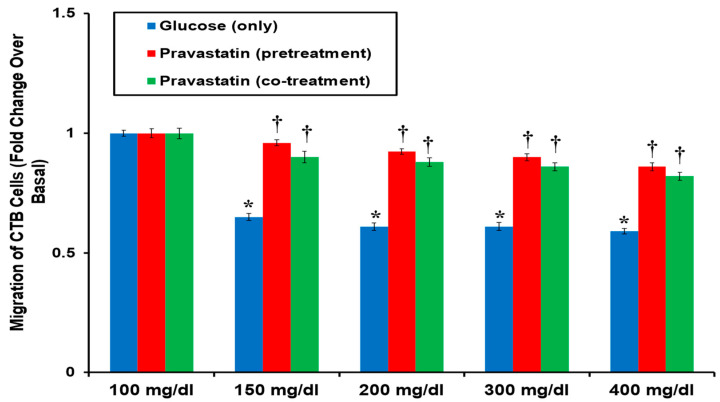
Both pravastatin pretreatment and cotreatment rescued hyperglycemia-induced CTB cell migration: Serum-starved CTB cells were treated with 100, 150, 200, 300, or 400 mg/dL of glucose (Sigma) for 48 h. Some cells were pretreated with 1 µg/mL of pravastatin before treatment with glucose, while others were cotreated with 1 µg/mL of pravastatin and the above glucose levels. All treated cells were subsequently added to transwell inserts that contained 10 ng/mL EGF and/or 100 ng/mL HGF. CTB cell migration was significantly (* *p* < 0.05) inhibited by ≥150 mg/dL of glucose that was significantly (* *p* < 0.05) attenuated by both pretreatment and cotreatment with 1.0 µg/mL pravastatin. Results are presented as mean ± SEM (*n* = 5, four replicates each). Both * and † are statistically significant.

Figure 4 shows that CTB cell migration was significantly inhibited by ≥150 mg/dL of glucose compared to basal glucose (100 mg/dL). The anti-migratory effect of glucose was attenuated by both pretreatment and cotreatment groups with 1.0 µg/mL pravastatin.

### 3.5. Effects of Pravastatin on Pathways of Abnormal Placentation

**Figure 5:**
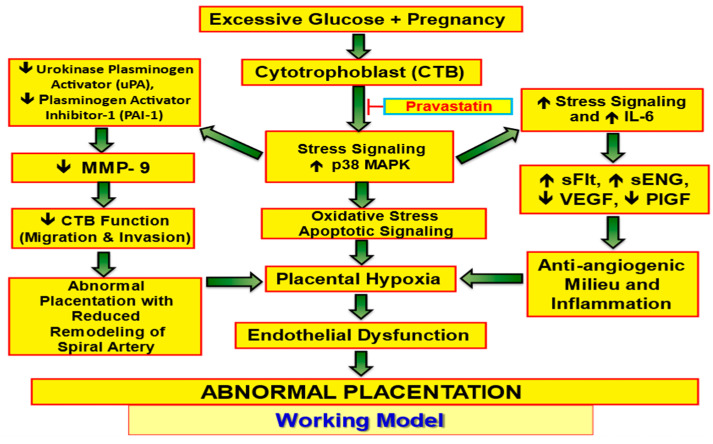
A new model summarizing the inhibitory effect of pravastatin on the stress-signaling pathway that leads to abnormal placentation.

Figure 5 shows a revised version of a prior model proposed by our prior research. We summarize the inhibitory effect of pravastatin on the stress-signaling pathway that leads to abnormal placentation.

## 4. Discussion

Numerous studies have determined that PE is often associated with inadequate endovascular cytotrophoblast (CTB) function [8,9,38,39]. We have previously shown that hyperglycemia acts as a stress signal by upregulating the p38/MAPK signaling pathways [40]. We also previously demonstrated that hyperglycemia disrupts the plasmin pathway through a dose–effect downregulation of uPA and PAI-1 [16]. Additionally, hyperglycemia-induced changes in angiogenic properties in CTB cells are consistent with those seen in the development of PE [10,11,37]. Pathology obtained from the placentas of diabetic pregnancies has demonstrated inadequate CTB invasion of the myometrium, further supporting this pathophysiology of PE [41]. The association of CTB cell disruption and hyperglycemia provides insight into how pregestational diabetes may predispose patients to a greater likelihood of developing preeclampsia. With this in mind, here we propose pravastatin as a potential prophylactic therapy for pregnant patients with pregestational diabetes to potentially decrease the risk of developing PE.

In this study, we induced hyperglycemia in CTB cells with appropriate concentrations of glucose to mimic human hyperglycemic conditions. The glucose concentrations used were 100, 150, 200, 300, and 400 mg/dL. In our hyperglycemia-induced, no-treatment control group, we used real-time PCR to show a signaling pathway consistent with that of CTB cells subjected to glucose insult, including downregulation of the mRNA expression of uPA/GAPDH and PA-1/GAPDH [10].

Real-time PCR is advantageous because all events and measurements are completed within the same testing tube, minimizing potential contaminants and increasing the ability to identify mRNA fragments during PCR processing.

A small observational prospective study (*n* = 21) revealed that subjects receiving treatment with Pravastatin 20 mg/day after diagnosis of PE showed improved blood pressure, improved uterine blood flow, and longer pregnancy duration [42]. However, the study was limited by the sample size and did not include the signaling profile associated with PE [42]. Another double-blind placebo-controlled randomized trial that analyzed the development of PE with the use of prophylactic pravastatin found no development of PE in the experimental group (*n* = 11) compared to the placebo (*n* = 10) [43]. While these studies show that pravastatin may attenuate the clinical development of PE, our study further investigates the potential benefits of pravastatin as pretreatment by showing attenuation of hyperglycemia-induced signaling pathways.

Gene expression analysis showed that both the Pravastatin pretreatment (>200 mg/dL glucose) and cotreatment (>300 mg/dL) groups resulted in upregulation in the expression of uPA and PAI-1. This indicates a decrease in the stress-signaling response that commonly leads to abnormal placentation and subsequent development of PE in pregnancy. These results were noted in PAI-1 regardless of glucose level. However, uPA was upregulated at a lower glucose level in the pretreated cells compared to those cotreated with pravastatin, suggesting that pravastatin pretreatment may be more effective for less severe cases of diabetes in pregnancy. In regard to the angiogenic growth factors implicated in PE development, we have previously shown glucose-induced downregulation in VEGF and PIGF and upregulation of sENG and sFLT-1 [10]. In this study, we determined that pravastatin prevented these changes by attenuating the downregulation of VEGF and PIGF and the upregulation of sENG and sFLT-1 at all supraphysiologic glucose levels. The statistically significant reduction in these signaling markers further supports our understanding of pravastatin’s ability to mitigate hyperglycemia-induced insult to placentation.

Hyperglycemia at >150 mg/dL showed a decrease in migration of CTB. Pravastatin pretreatment and cotreatment attenuated this hyperglycemia-induced decrease in migration, suggesting that pravastatin may improve placentation by allowing normal CTB migration and invasion and thereby reducing the risk of PE [8,9,38,39]. Our results support the hypothesis that it may be advantageous to utilize pravastatin in pregnancies complicated by hyperglycemia to mitigate and potentially decrease the risk of PE development by inhibiting the hyperglycemia-induced signaling pathways that facilitate the development of PE.

Although our study supports pravastatin as a potential prophylactic therapy, further research needs to be conducted to prove its safety and efficacy before it can be implemented. The two clinical trials will provide insight into these characteristics, as will our continued research into the relationship between diabetes and PE. This will further expand our knowledge of the disease process of PE and help develop effective methods for disease prevention in high-risk populations.

## Data Availability

Data are contained within the article.
